# Supplementary material for: Detection of companion galaxies around hot dust-obscured hyper-luminous galaxy W0410-0913
Source: Nat Commun. 2022 Aug 5;13:4574. doi: 10.1038/s41467-022-32297-x (PMC9355969; doi:10.1038/s41467-022-32297-x)
Supplement: Supplementary file 1 — Supplementary Information [file 41467_2022_32297_MOESM1_ESM.pdf]

Supplementary Information: **Detection of companion galaxies around hot dust-obscured hyper-luminous galaxy W0410-0913**

M. Ginolfi et al.

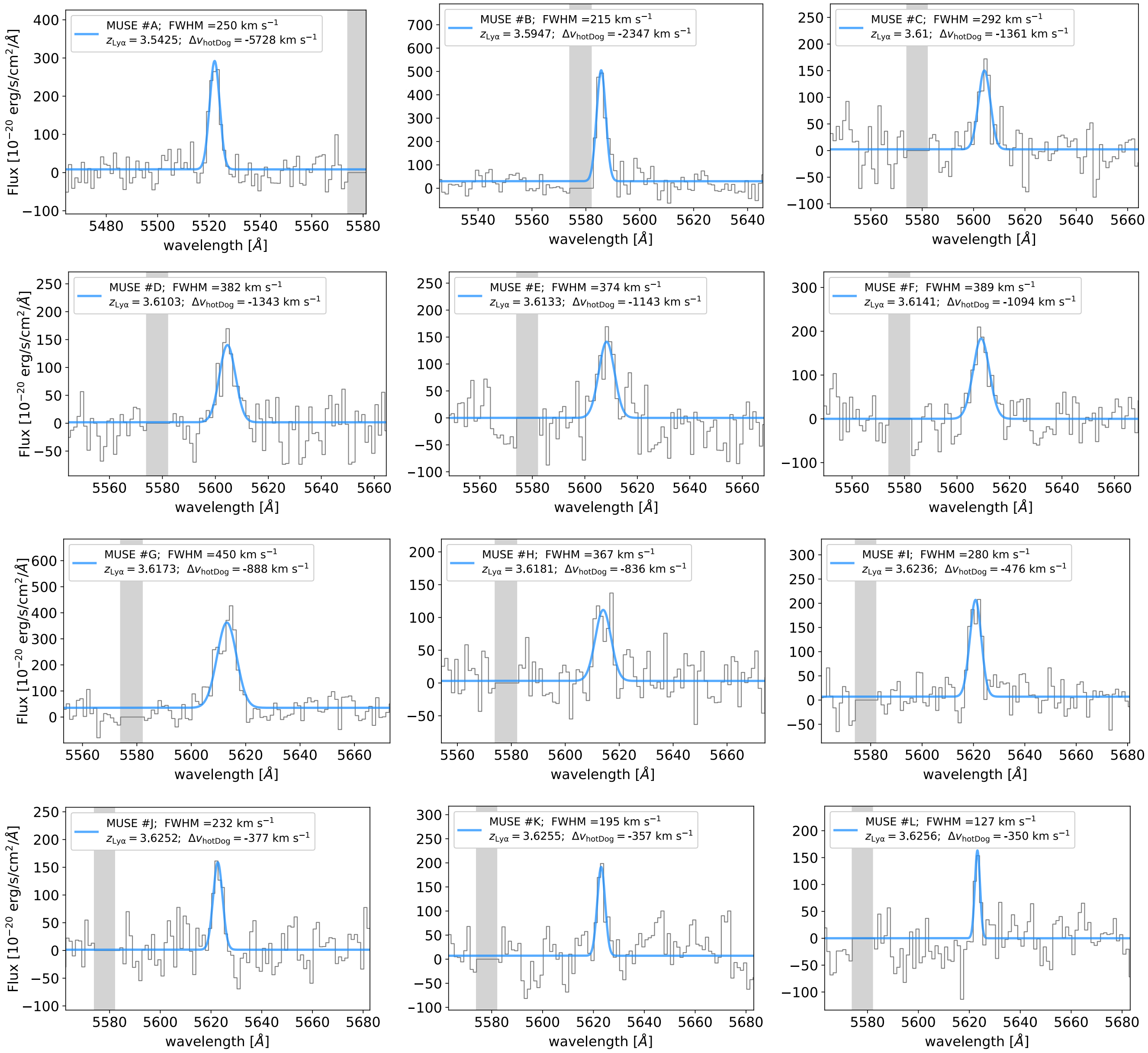

Supplementary Figure 1. Lyman-α spectra of the companion galaxies discovered with MUSE in the surrounding environment of W0410-0913. Lyman-α spectra, extracted from circular regions with a radius of 1″ and centered around the Lyman-α peaks, are shown in gray. The wavelength range in the x-axis has a width of 120 Å and is centered around the central wavelength obtained through a Gaussian fit (Gaussian models are shown in blue). Legends report the redshift of each source (derived through Lyman-α), their FWHM, and their offset velocity with respect to the systemic redshift of the central luminous object. Gray shaded areas represent the regions affected by sky lines. See Table 1 for the definition of IDs and a summary of the measured properties of the Lyman-α emitters. Figure continues in Supplementary Figure 2.

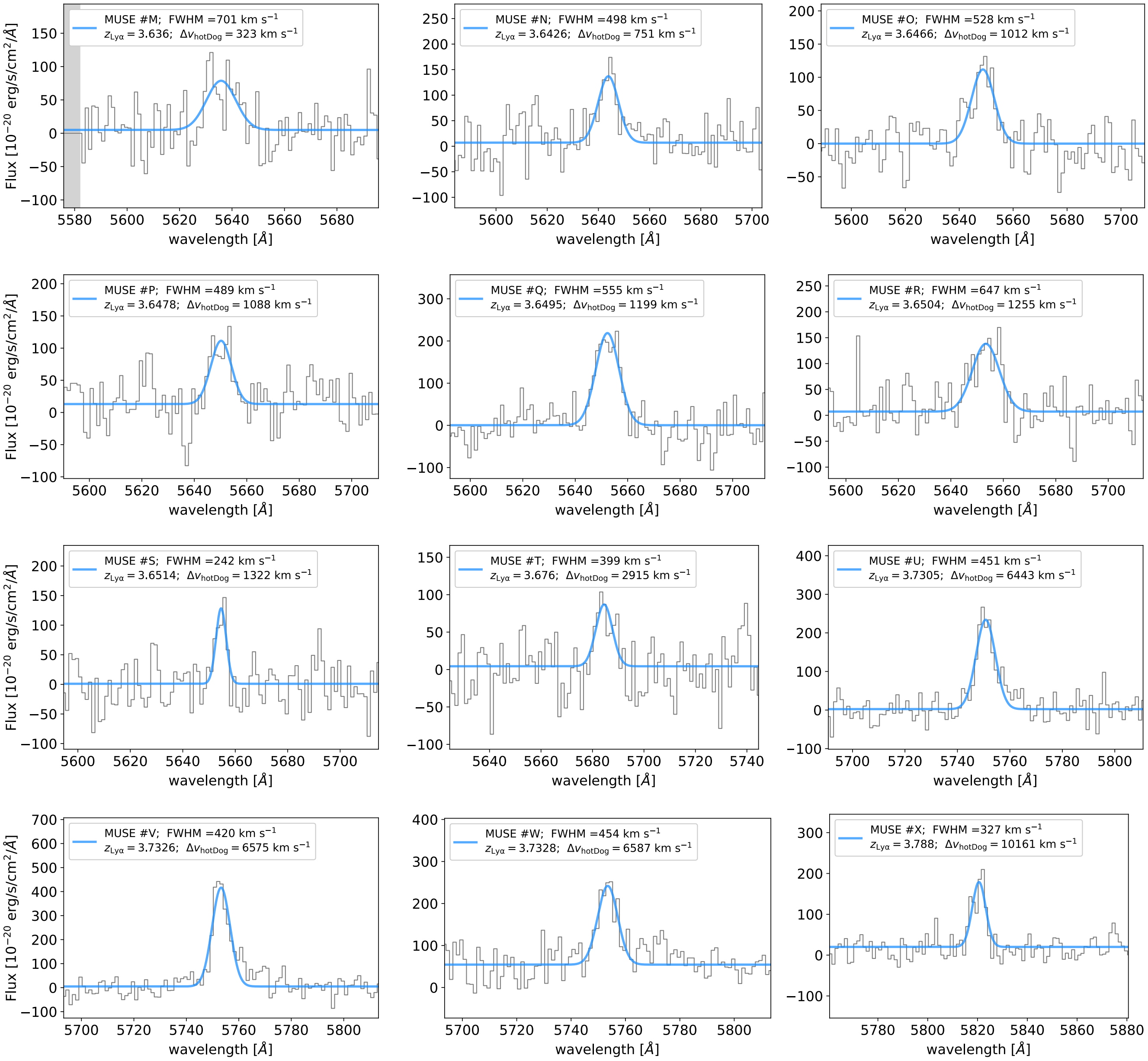

Supplementary Figure 2. Lyman-α spectra of the companion galaxies discovered with MUSE in the surrounding environment of W0410-0913. Lyman-α spectra, extracted from circular regions with a radius of 1″ and centered around the Lyman-α peaks, are shown in gray. The wavelength range in the x-axis has a width of 120 Å and is centered around the central wavelength obtained through a Gaussian fit (Gaussian models are shown in blue). Legends report the redshift of each source (derived through Lyman-α), their FWHM, and their offset velocity with respect to the systemic redshift of the central central luminous object. Gray shaded areas represent the regions affected by sky lines. See Table 1 for the definition of IDs and a summary of the measured properties of the Lyman-α emitters. Figure starts in Supplementary Figure 1.

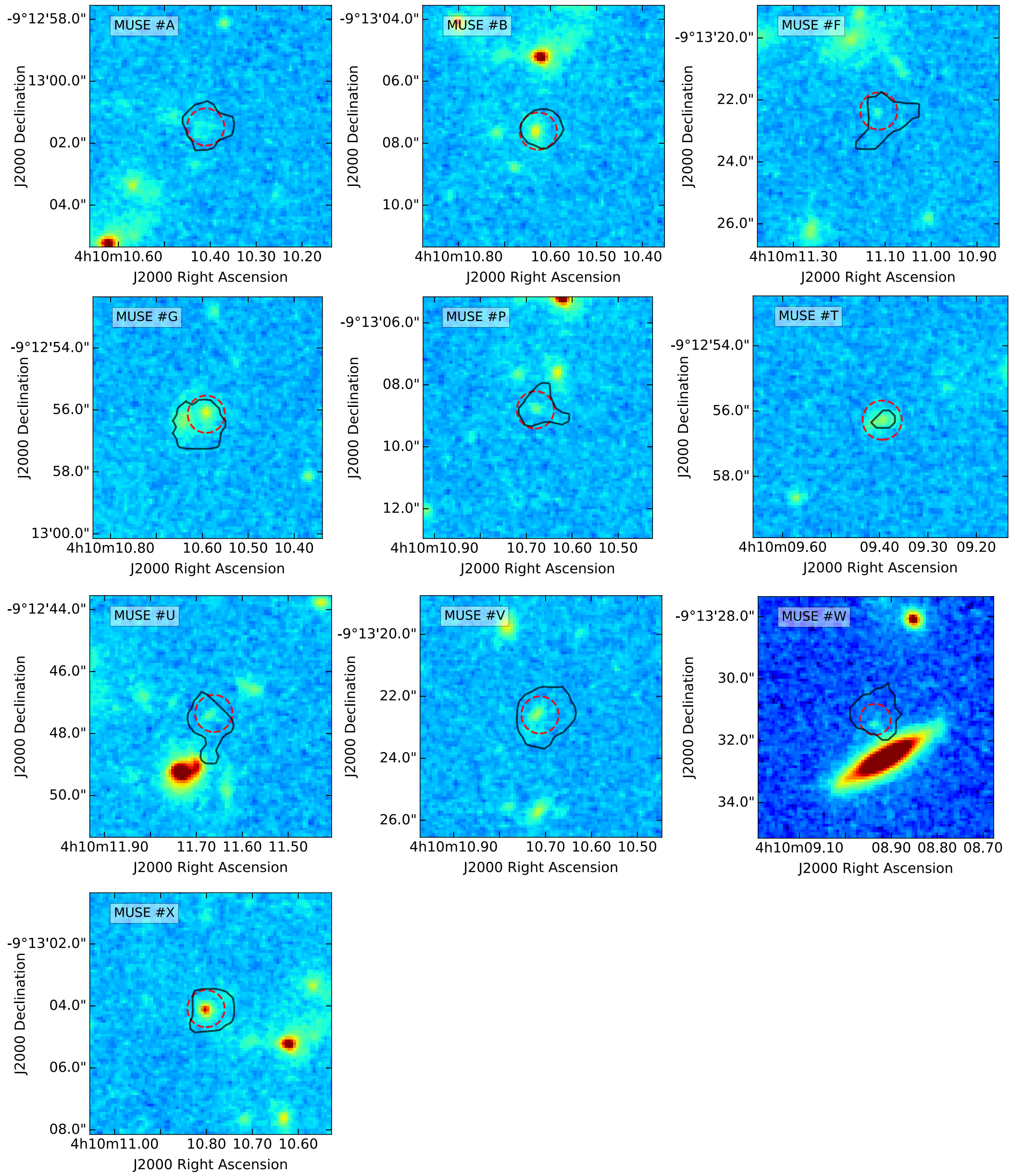

Supplementary Figure 3. Maps of the HST-detected companion galaxies. The HST cutouts ($4'' \times 4''$) of the Lyman-α emitters detected with the WFC3/F160W filter are shown (colored images). The 2σ contours of pseudo-NB Lyman-α images obtained with MUSE are overlaid in black. Red dashed circles represent the circular apertures with $1''$-sized radii that we use to extract the HST flux and estimate the SFR.
